# Direct Arylation of 1,10-Phenanthroline Using an Organomagnesium Reagent

**DOI:** 10.1021/acsomega.5c07575

**Published:** 2025-11-13

**Authors:** Nathanael A. Hirscher, Julian B. O. Van Esselstine, Bryan C. Yapo, Nadia Asif Masood, Erika L. Vandooijeweert

**Affiliations:** Department of Chemistry and Biochemistry, 8087 Montclair State University, 1 Normal Ave., Montclair, New Jersey 07043, United States

## Abstract

Substituted derivatives of 1,10-phenanthroline are important ligands in inorganic chemistry. Such ligands are often synthesized by nucleophilic addition of aryl- or alkyllithium reagents to the carbon positions adjacent to the phenanthroline nitrogens. The typical reaction sequence (organolithium addition, protonolysis, oxidation, and rearomatization) has been widely used and seldom improved. We sought to replace lithium-based reagents with safer reagents based on magnesium (i.e., Grignards). The effective use of an arylmagnesium reagent in the production of a phenanthroline ligand derivative is now disclosed. Additionally, our newly reported method avoids the use of a heterogeneous oxidant (MnO_2_) which the traditional method employed in excess. Instead, oxidation due to air exposure is used to achieve the final aromatization step. This new reaction scheme produces 2,9-di­(*p*-methoxyphenyl)-1,10-phenanthroline in a process with a 10.2% average yield.

## Graphical abstract

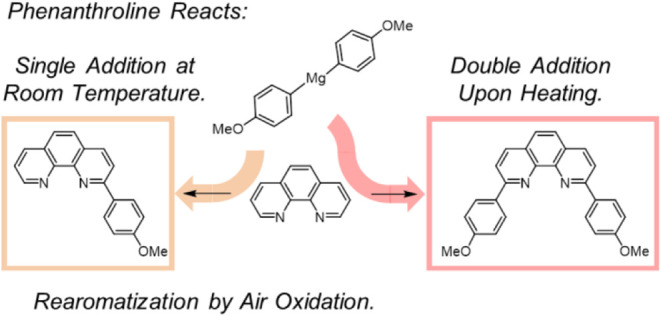

## Introduction

The 1,10-phenanthroline ligand (C_12_H_8_N_2_, or phenanthroline from here on) is used as a colorimetric indicator due to its strong binding to transition metals, generating light-absorbing complexes.^1^ Phenanthroline is also an auxiliary ligand for transition-metal catalysts in photochemical applications.^2^ Synthetic modifications to phenanthroline by substitution at the planar, tricyclic core have led to numerous derivatives.^3^ The metal-binding ability of the phenanthroline nitrogens ultimately underpins many of these applications. The proximity of the 2 and 9 carbons to these metal-binding nitrogens makes these “*ortho*” positions extremely attractive sites for derivatization.^4,5^ Indeed, the seminal report on the nucleophilic substitution of phenanthroline using aryllithiums by Dietrich-Buchecker and Sauvage (Scheme 1, top) was parlayed into elegant work on supramolecular structures.^6–8^ Besides the double addition of aryllithiums, a variety of other routes to 2,9-disubstituted phenanthrolines have been developed. One alternative route is alkylation using radical precursors activated by Ir photoredox catalysts.^9^ A popular modern route is via Pd-catalyzed cross-coupling.^10–14^ The photoredox route seems convenient for the installation of alkyl substituents but is not yet applicable for aryl groups. The cross-coupling route depends on a multistep process to prechlorinate the phenanthroline core, generating substantial chemical waste.

**Scheme 1:**
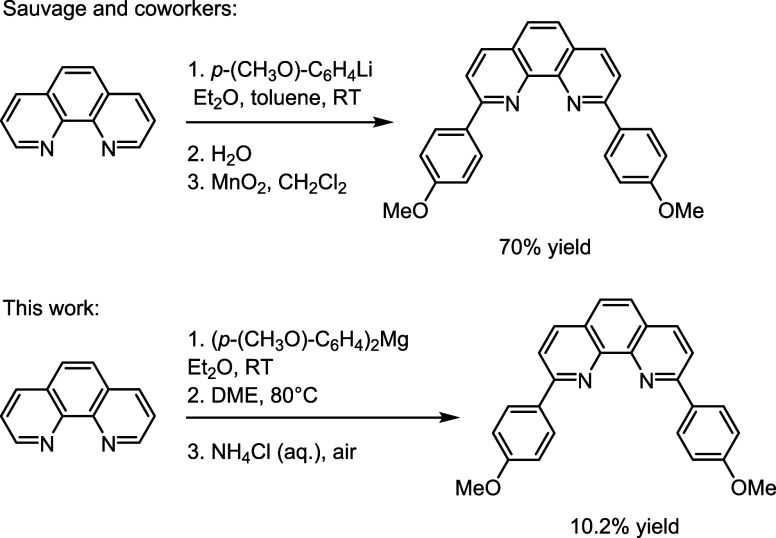
Top: Traditional Synthesis of Disubstituted Phenanthroline Using an Aryllithium Reagent (Refs 6,15 ). Bottom: Our Synthesis Using an Arylmagnesium Reagent. DME = 1,2-Dimethoxyethane

Inspired by the simplicity and utility of Sauvage’s original synthesis of 2,9-dianisyl-1,10-phenanthroline (dap, or 2,9-di­(*p*-methoxyphenyl)-1,10-phenanthroline),^6,15^ we imagined the use of Grignard reagents as nucleophiles instead of the more reactive aryllithiums (see Scheme 1). The resulting ligand in question (dap) has been utilized by numerous researchers to support photoactive, catalytic copper compounds.^11,12,16–20^

The direct arylation of 1,10-phenanthroline with magnesium-based reagents has not been reported. In the patent literature, an aryl-Grignard was reacted with substituted phenanthrolines (but notably—not unsubstituted phenanthroline).^21,22^ Halogenated phenanthrolines were demonstrated to be susceptible to the same kind of direct substitution by Grignards, but this also required prefunctionalization.^23,24^ Grignard reagents were used in the substitution of quinolines and other heteroaromatics (but notably—not any phenanthroline).^25^

In the context of the importance of substituted phenanthrolines in organometallic chemistry, as well as the widespread use of phenanthroline as a colorimetric indicator for Grignards,^26,27^ we undertook our synthetic investigation. We report here that ortho-addition to phenanthroline readily occurs using an anisylmagnesium reagent (anisyl = *p*-methoxyphenyl) at room temperature. A second ortho-addition requires heating. This reactivity was successfully used to achieve a double substitution of phenanthroline to generate 2,9-di-(*p*-methoxyphenyl)-1,10-phenanthroline (see Scheme 1, bottom). Notably, we have eliminated the need for organolithium reagents by using an organomagnesium reagent instead. Therefore, our new methodology is potentially a safer and more convenient route to 2,9-disubstituted 1,10-phenanthrolines compared to existing procedures (see Scheme 2).

**Scheme 2:**
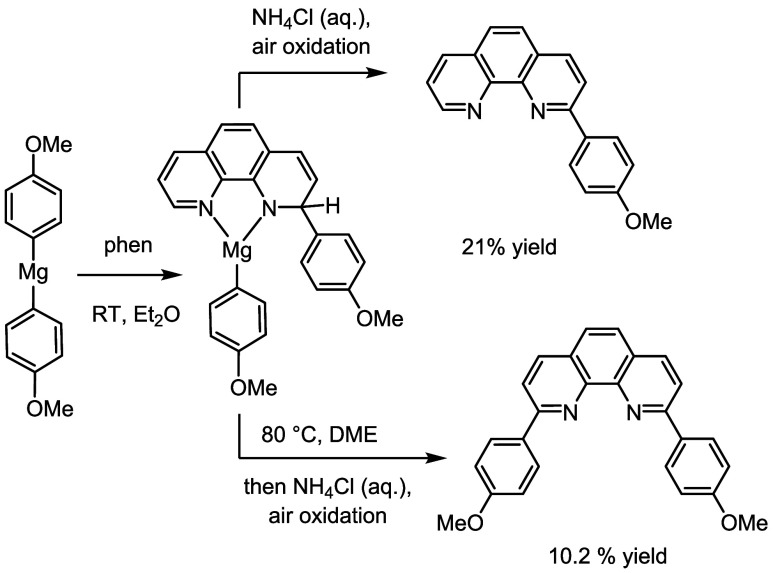
We Propose that a Monosubstituted Intermediate Forms at Room Temperature (RT). Quenching and Oxidation (With No Heating) Lead to 2-(*p*-Methoxyphenyl)-1,10-phenanthroline (Top). Subsequent Heating before Quenching and Oxidation Leads to the Second Anisyl addition (Bottom)

## Results and Discussion

The anisyl Grignard complex (*p*-methoxyphenyl magnesium bromide) is readily generated by mixing 4-bromoanisole with Mg turnings in dry tetrahydrofuran (THF) or dry Et_2_O. Our initial exploration utilized Grignard solutions in THF, but we switched to Et_2_O due to selectivity problems in THF (*vide infra*). Interestingly, in Et_2_O, the *p*-methoxyphenyl magnesium bromide is only partially soluble: a gray oil separates. This behavior seemed unusual to us, as it was not accompanied by any solid precipitation, which would be indicative of MgBr_2_ formation. Due to the difficulty of working with concentrated mixtures of the Grignard (i.e., *p*-(CH_3_O)-C_6_H_4_MgBr) in Et_2_O, the Schlenk equilibrium was intentionally induced by the addition of 1,4-dioxane.^28^ Filtration and/or decantation of the resulting supernatant away from white, solid MgBr_2_ yielded homogeneous solutions we formulate as containing (*p*-(CH_3_O)-C_6_H_4_)_2_Mg (i.e., dianisylmagnesium). This diaryl magnesium reagent was titrated against menthol to provide the concentration of the nucleophilic aryl group. The reactions discussed here utilize the (*p*-(CH_3_O)-C_6_H_4_)_2_Mg reagent, which is solvated effectively by Et_2_O.

Upon the addition of the (*p*-(CH_3_O)-C_6_H_4_)_2_Mg reagent to solid phenanthroline under air-free conditions, a dark red-brown suspension resulted. After stirring at RT and then protic quenching (aqueous NH_4_Cl), oxidation was performed by prolonged stirring in the presence of air. From ^1^H NMR analysis of the crude mixture, 2-(*p*-methoxyphenyl)-1,10-phenanthroline was observed to be the major product. That is, the nucleophilic addition only occurs once at room temperature. Following this method, the monosubstituted anisyl phenanthroline (map or 2-(*p*-methoxyphenyl)-1,10-phenanthroline) could be obtained in pure form in a 21% average yield (see Supporting Information). Less attention has been paid to synthetic methods of monosubstituted phenanthrolines,^29^ but control over the addition rate by convenient temperature control may streamline the synthesis of asymmetric phenanthroline derivatives.^30,31^

Heating the mixture of 1,10-phenanthroline and (*p*-(CH_3_O)-C_6_H_4_)_2_Mg required a higher-boiling solvent. This was done conveniently by evaporation of Et_2_O under vacuum and then addition of dry 1,2-dimethoxyethane (DME). Heating to 80 °C then led to the same sequence of protic quenching and air oxidation, leading to the formation of 2,9-di­(*p*-methoxyphenyl)-1,10-phenanthroline as the major product. A lengthy purification scheme was required for this compound: aqueous-organic extraction, column chromatography, and a final recrystallization yielded the pure compound in a 10.2% average yield (see Supporting Information). The low yield indicates the existence of side reactions, one of which is the “back addition” of the aryl group, which is discussed below. No unreacted phenanthroline (and only small amounts of monosubstituted product) were observed in the crude reaction mixtures following heating.

We hypothesize that the arylmagnesium reacts in a typical nucleophilic fashion, in analogy to the proposed mechanism using aryllithiums.^15^ The requirement for a higher temperature to induce the second aryl addition is likely a consequence of the buildup of an additional negative charge in the intermediate. Protonolysis of the anionic intermediates leads to dearomatized intermediates that need to be oxidized to restore aromaticity, yielding the final phenanthroline products.

For both mono- and disubstituted products, oxidative rearomatization occurred by stirring in the presence of air. The oxidation was done by stirring the crude mixtures open to the laboratory atmosphere immediately following the protonolysis (i.e., before the aqueous–organic extraction step). Thus, the oxidation occurred in a biphasic Et_2_O/water mixture for the monosubstituted product and in a DME/water mixture for the disubstituted product. In this fashion, we have avoided the use of excess MnO_2_ that was the typical oxidant in the related procedures developed by Sauvage and coworkers. In our experience, MnO_2_ is a particulate solid that is prone to clogging a filtration apparatus and leads to substantial chemical waste.

Our preliminary efforts to expand the scope of this reactivity have suggested that the aryl structure plays a key role. We moved from the successful dianisylmagnesium to a reagent with a bulky steric profile, (2,4,6-trimethylphenyl)_2_Mg (dimesitylmagnesium).^32^ No evidence of phenanthroline substitution by dimesitylmagnesium at room temperature was seen—contrasting with the monosubstitution seen for dianisylmagnesium. Possibly, there is steric inhibition of aryl group migration from a (phen)­MgR_2_ (*R* = aryl) complex when *R* = mesityl. Notably, the organolithium chemistry is successful for mesityl addition to phenanthroline.^33^ Monoarylation using (*o*-tolyl)_2_Mg at RT was performed, leading to 2-(*o*-tolyl)-1,10-phenanthroline.^34^ Thus, phenanthroline does react with other arylmagnesium reagents (see Supporting Information for details). Further studies will be required to deconvolute the steric vs electronic factors playing into the difference in reactivity based on the aryl nucleophile structure.

As mentioned earlier, the selection of the ethereal solvent is important. The final synthetic procedures reported herein use Et_2_O as a solvent (for room temperature reactions) and DME (for reactions at elevated temperature). In contrast, (*p*-methoxyphenyl)­MgBr was prepared in THF and used as the reagent for the direct arylation of phenanthroline at room temperature. The resulting major species (following oxidation) was 4-(*p*-methoxyphenyl)-1,10-phenanthroline,^35^ a regioisomer which corresponds to undesired arylation at the back of the phenanthroline core. To further establish that THF leads to erosion of selectivity (as opposed to effects of Grignard speciation), the (*p*-methoxyphenyl)_2_Mg reagent was added to a THF solution of 1,10-phenanthroline (see Supporting Information for details). The resulting crude mixture contained a 1.6:1 ratio of “*para*” and “*ortho*” isomers (4-(*p*-methoxyphenyl)-1,10-phenanthroline and 2-(*p*-methoxyphenyl)-1,10-phenanthroline). The mechanistic role of the solvent in directing the regioselectivity of arylation is still an open question. Notably, the requirement for a highly selective process is more stringent when a second arylation is desired.

## Conclusion

A widely used phenanthroline derivative was synthesized by a new procedure. 2,9-Di­(*p*-methoxyphenyl)-1,10-phenanthroline was made without using lithium-based reagents or expensive transition metal (Ir or Pd) catalysts. Instead, arylmagnesium reagents were found to be sufficiently reactive for the addition reaction. Monosubstitution occurs readily at room temperature, whereas disubstitution requires heating to 80 °C. Following a protic quench, oxidative rearomatization was done using air, without the need for MnO_2_ or other reagents. Overall, this procedure is a safer and more convenient direct synthesis of a diaryl phenanthroline derivative compared to existing procedures.

## Experimental Section

For general considerations, additional synthetic procedures, and NMR spectra, see Supporting Information.

### Synthesis of 2,9-Di­(*p*-methoxyphenyl)-1,10-phenanthroline

To a 500 mL Schlenk flask were added Mg turnings (3.01 g, 0.124 mol) along with a stir bar. The flask was heat-gunned under vacuum, then backfilled with N_2_, and allowed to cool to RT. Using a syringe, 48 mL of diethyl ether was added. Next, 3 mL of 4-bromoanisole was added all at once by using a syringe. The mixture was stirred vigorously until there was evidence of Grignard initiation: the solution became cloudy. Over the course of 1 h, ≈2 mL portions of 4-bromoanisole were added to maintain an exothermic reaction. After all 4-bromoanisole was added (total amount of 4-bromoanisole added was 15 mL, or 0.12 mol), the mixture was brown in color, and most of the Mg turnings were obviously consumed. After stirring for 30 min further, 5.4 mL (0.063 mol) of 1,4-dioxane was added portionwise over 20 min. White precipitate quickly formed upon the first drops, and stirring was maintained throughout the addition of the 1,4-dioxane. The thick slurry of magnesium salts was stirred for 18 h at room temperature. Then, 96 mL of Et_2_O was added to dilute the supernatant. The mixture was stirred for several more minutes, and then, the solids were allowed to settle. The supernatant was decanted by cannula transfer (aided by a cannula filter) into a dry, degassed Schlenk tube under N_2_, and 48 mL Et_2_O was added to the original suspension to repeat the decantation process. All of the decanted supernatant amounted to approximately 120 mL of a homogeneous yellow solution, and this was stored to await titration.

Titration of dianisylmagnesium was performed according to the following procedure.^26,27^ An oven-dried, 3-neck flask equipped with a stir bar was placed under N_2_ on the Schlenk line. Then, against a counterflow of N_2_, (−)-menthol (0.067 g) was added, along with 1,10-phenanthroline (≈0.004 g) used as an indicator. Approximately 5 mL of dry THF was added via a syringe. The colorless, homogeneous solution was stirred at RT, and then, dianisylmagnesium solution was added dropwise using a 3 mL plastic syringe affixed to a long metal needle to an end point (0.90 mL) marked by an opaque, pink solution. This corresponded to an organometallic anisyl (i.e., active Brønsted base) concentration of 0.48 M. Note: the titration value used here is irrespective of Grignard speciation (R_2_Mg vs RMgBr—although we suppose it to be in the diorganomagnesium form) in which case the value should be referred to as the R_2_Mg *normality* (0.48 N).

A separate 500 mL Schlenk tube was charged with 1,10-phenanthroline (2.647 g, 0.0147 mol) and a stir bar. This flask was evacuated for 2 h on the Schlenk line. Then, it was backfilled with N_2_. The titrated dianisylmagnesium solution (anisyl concentration = 0.48 M) was added via syringe transfer (120.5 mL, 0.058 mol of anisyl) to the solid phenanthroline. The resulting dark red-purple suspension was stirred at RT for 20 h. Then, Et_2_O was removed *in vacuo* using the Schlenk line. The resulting brown solid was dissolved in 88 mL of dry 1,2-dimethoxyethane (DME). The mixture was heated to 80 °C in an oil bath for 22 h. After this time, the reaction was cooled to RT, and then, NH_4_Cl (7.84 g, 0.147 mol) in 100 mL of H_2_O was used to quench the reaction via slow addition (30 min). A prompt color change to yellow occurred. The entire mixture was poured into a wide-mouth recrystallization dish and stirred—open to the atmosphere—for 18 h. The progress of the reaction can be judged qualitatively by the density/morphology of the organic phase. Before air oxidation, low-density orange droplets floated on the aqueous phase. Afterward, orange solid (or oily liquid) was at the bottom of the dish.

To recover the crude product, 100 mL of CH_2_Cl_2_ and 50 mL H_2_O were added, and the mixture was transferred to a separatory funnel. The organic layer was separated, and the aqueous layer was extracted with 2 × 50 mL of CH_2_Cl_2_. The combined organic layers were dried with MgSO_4_ and filtered. The crude organic mass was obtained (5.75 g) after evaporation of the solvent and purified by column chromatography. Column diameter: 6 cm. Column height: 16 cm. Eluent: 1:2 EtOAc:hexanes. *R*_*f*_ (1:2 EtOAc:hexanes) = 0.5. A coeluting impurity was removed by recrystallization from hot toluene. The pure product was obtained after drying under vacuum (<100 mTorr) at 150 °C to yield a yellow solid (0.579 g, 10.0% yield). Average yield across three trials (see Supporting Information): 10.2%. ^1^H NMR (400 MHz, CDCl_3_): δ 8.43 (d, *J*_HH_ = 8.6 Hz, 4H), 8.25 (d, *J*_HH_ = 8.4 Hz, 2H), 8.08 (d, *J*_HH_ = 8.4 Hz, 2H), 7.72 (s, 2H), 7.11 (d, *J*_HH_ = 8.6 Hz, 4H), 3.92 (s, 6H). ^13^C {^1^H} NMR (100.7 MHz, CDCl_3_): δ 161.0, 156.4, 146.1, 136.9, 132.3, 129.1, 127.7, 125.7, 119.5, 114.3, 55.5. Elemental Analysis Calcd (%) for C_26_H_20_N_2_O_2_: C, 79.57; H, 5.14; N, 7.14. Found (%): C, 79.06; H, 5.32; N, 6.77. EI-MS (*m*/*z*): 392.12.

## Supplementary Material



## Notes

General considerations, additional synthetic procedures, and NMR spectra (PDF)
