# Supplementary material for: Direct Arylation of 1,10-Phenanthroline Using an Organomagnesium Reagent
Source: ACS Omega. 2025 Nov 13;10(46):55989–93. doi: 10.1021/acsomega.5c07575 (PMC12658829; doi:10.1021/acsomega.5c07575)
Supplement: Supplementary file 1 [file ao5c07575_si_001.pdf]

Electronic Supporting Information.

Direct Arylation of 1,10-Phenanthroline Using an Organomagnesium Reagent.

Nathanael A. Hirscher*, Julian B. O. Van Esselstine, Bryan C. Yapo, Nadia Asif Masood, and Erika L. Vandooijeweert

Author affiliations: Department of Chemistry and Biochemistry, Montclair State University, 1 Normal Ave. Montclair, NJ 07043.

*To whom correspondence should be addressed: hirschern@montclair.edu

Table of Contents.

General Considerations………………………………………………………………… Page S2

Synthesis of 2-(*p*-methoxyphenyl)-1,10-phenanthroline…………………………... Page S3

Replication of Synthetic Trials……………………………………........................ Page S4

NMR Characterization Data……………………………………………………… Page S5

UV-Vis Data………………………………………………………………………… Page S7

GC/MS Data…………………..…………………………………………………. Page S9

Attempted Arylation with Dimesitylmagnesium……………………………………. Page S11

Successful Arylation with Di(*o*-tolyl)magnesium…………………………………. Page S12

Arylation with (*p*-methoxyphenyl)MgBr in THF……………………………………. Page S13

Arylation with (*p*-methoxyphenyl)$_2$Mg in THF……….……………………………. Page S13

References………………………………………………………………………… Page S15

General Considerations.

All synthetic procedures involving organomagnesium reactants/products were performed in oven-dried glassware (unless otherwise specified) using standard Schlenk techniques (or in the glovebox—where specified) under an atmosphere of N_2. Solvents 1,2-dimethoxyethane (TCI America) and tetrahydrofuran (TCI America) were dried over sodium and distilled prior to use. For reactions performed in the glovebox, Et_2O (Thermo Scientific Chemicals) and 1,4-dioxane (TCI America) were each dried over sodium and distilled prior to use. For the synthetic procedures using the Schlenk line, Et_2O and 1,4-dioxane were purchased from Thermo Scientific Chemicals, stored in Acroseal bottles over molecular sieves, and removed by syringe transfer. Dichloromethane, toluene, ethyl acetate, and hexanes were purchased from Fisher Chemical. Reagents 4-bromoanisole (97.0+%, TCI America), (-)-menthol (99.0+%, TCI America), NH_4Cl (98+% Thermo Scientific Chemicals), and anhydrous $MgSO_4$ (97% Thermo Scientific Chemicals) were purchased from commercial sources and used without further purification. The 2-bromomesitylene (99%, Thermo Scientific Chemicals) and 2-bromotoluene (98.0+%, TCI America) were vacuum distilled before use in the glovebox. The 1,10-phenanthroline (Fisher Scientific Chemicals, 99%, may contain up to 1.5% water) was dried under vacuum overnight before use in the glovebox, otherwise it was dried under vacuum for 2 hr. Magnesium turnings (99+%) and manganese oxide (activated, technical grade) were purchased from Thermo Scientific Chemicals and used as received. Silica gel (60A) was purchased from Thermo Scientific Chemicals. Deuterated chloroform ($CDCl_3$) was purchased as 99.8% D from Cambridge Isotope Laboratories as used as received. Proton and carbon nuclear magnetic resonance spectroscopy (^1H and ^{13}C NMR) was performed on a Bruker Spectrospin Avance II 400 MHz Spectrometer. ^1H NMR chemical shifts were referenced to the solvent residual (typically $CHCl_3$ at 7.26 ppm relative to TMS). The ^{13}C NMR chemical shifts were referenced to the solvent (typically $CDCl_3$ at 77.16 ppm relative to TMS). UV-Vis data were collected on a Cary 60 Spectrophotometer (G6860A) with solutions contained in special optical glass cuvettes. The absorbance of a blank solvent sample was subtracted from the sample to generate the reported spectra. Elemental analysis was performed by Robertson Microlit Laboratories (Ledgewood, NJ). Gas chromatography-Mass spectrometry data was collected using an Agilent 8890/5977C GC/MS, using He carrier gas, electron ionization (EI), and single quadrupole mass spectrometric detection.

Synthesis of 2-(*p*-methoxyphenyl)-1,10-phenanthroline. In the glovebox, 3.017 g (0.0167 mol) of phenanthroline was added to a round-bottom flask along with a stirbar. To this, 100 mL of dianisylmagnesium (0.25 N in Et_2O, prepared and titrated against (-)-menthol as described in the main text, 0.025 mol anisyl) was added all at once. The flask was covered with a septum and the dark suspension was stirred for 5 days at RT. Then, it was taken out of the glovebox and quenched by dropwise addition of 2.697 g (0.0504 mol) of NH_4Cl dissolved in 20 mL H_2O. The resulting yellow mixture was left stirring, open to air, in the round bottom flask (with a 24/40 size neck) for three days. Then, ≈ 200 mL of CH_2Cl_2 was added, along with ≈ 80 mL H_2O. The aqueous layer was extracted with 3 x 25 mL of CH_2Cl_2. All organic layers were combined, dried over $MgSO_4$, and then filtered through paper. The filtrate was reduced to dryness *in vacuo* to obtain a brown oil. This oil was purified by column chromatography (eluent: 2:1 EtOAc:hexanes). R_f (2:1 EtOAc:hexane) = 0.4. After evaporation of the column eluent, a yellow solid was obtained. Further purification of the solid by sublimation (<100 mTorr, 140°C) was used to separate the product from unknown non-volatile impurities. This yielded a pale yellow powder: (0.797 g, 16.6% yield). ^1H NMR (400 MHz, $CDCl_3$): δ 9.22 (dd, J_{HH} = 4.5, 1.7 Hz, 1H), 8.32 (d, J_{HH} = 8.7 Hz, 2H), 8.23 (m, 2H), 8.03 (d, J_{HH} = 8.3 Hz, 1H), 7.76 (d, J_{HH} = 8.6 Hz, 1H), 7.71 (d, J_{HH} = 8.6 Hz, 1H), 7.61 (dd, J_{HH} = 7.9, 4.6 Hz, 1H), 7.06 (d, J_{HH} = 8.7 Hz, 2H), 3.88 (s, 3H). ^{13}C {^1H} NMR (100.7 MHz, $CDCl_3$): δ 161.0, 157.2, 150.2, 146.3, 146.0, 136.8, 136.3, 132.2, 129.4, 129.2, 127.2, 126.5, 125.8, 122.9, 120.1, 114.2, 55.5. Elemental Analysis Calcd. (%) for $C_{19}H_{14}N_2O$: C, 79.70; H, 4.93; N, 9.78. Found (%): C, 78.75; H, 5.14; N, 9.43. EI-MS (m/z): 286.08.

Replication of Synthetic Trials. The average yields reported in the main text of the manuscript were determined from the trials listed below. Each synthesis was performed in triplicate, with deviations from the detailed standard procedure of potential relevance indicated. Notably, the di-substituted product was synthesized on the Schlenk line and the mono-substituted product was synthesized in the glovebox. The need to heat the reaction for disubstitution necessitated running it on the Schlenk line. The mono-arylation runs only at room temperature and this is conveniently done in the glovebox.

Table S1. A list of synthetic trials used to calculate the reported average yields.

Product:	% Yield / Scale (mmol)	Deviation from Standard Procedure
2,9-di-(*p*-methoxyphenyl)-1,10-phenanthroline	10.0% / 14.7	N/A (see main text)
2,9-di-(*p*-methoxyphenyl)-1,10-phenanthroline	7.2% / 13.3	Mg(anisyl)$_2$ stirred for 4 days after dioxane addition (instead of 18 h). The Mg(anisyl)$_2$ and phen was stirred in Et_2O for 6 days at RT (instead of 20 h) before heating in DME.
2,9-di-(*p*-methoxyphenyl)-1,10-phenanthroline	13.3% / 10.4	None
2-(*p*-methoxyphenyl)-1,10-phenanthroline	16.6% / 16.7	N/A (see above section)
2-(*p*-methoxyphenyl)-1,10-phenanthroline	13.4% / 16.6	The Mg(anisyl)$_2$ and phen was stirred in Et_2O for 6 days at RT (instead of 5 days)
2-(*p*-methoxyphenyl)-1,10-phenanthroline	32.5% / 16.9	No sublimation required after column. Mg(anisyl)$_2$ solution prepared using 4 equiv. 1,4-dioxane (instead of 0.5 equiv.).
2-(*p*-methoxyphenyl)-1,10-phenanthroline	n/a*	Mg(anisyl)$_2$ and phen stirring at RT for 1 day.

*No yield was determined but the reaction mixture contained primarily unreacted phenanthroline. It is possible that the stirring rate was insufficient to mix the suspension.

NMR Characterization Data.

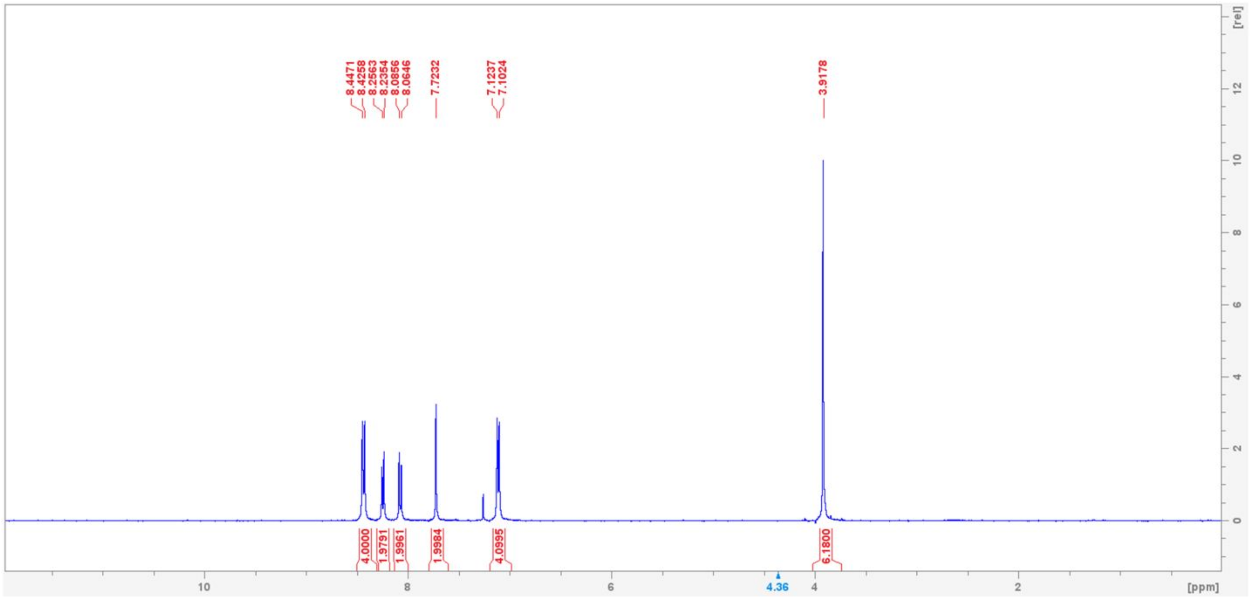

Figure S1. ^{1}H NMR ($CDCl_3$, 400 MHz) spectrum of 2,9-di-(*p*-methoxyphenyl)-1,10-phenanthroline.

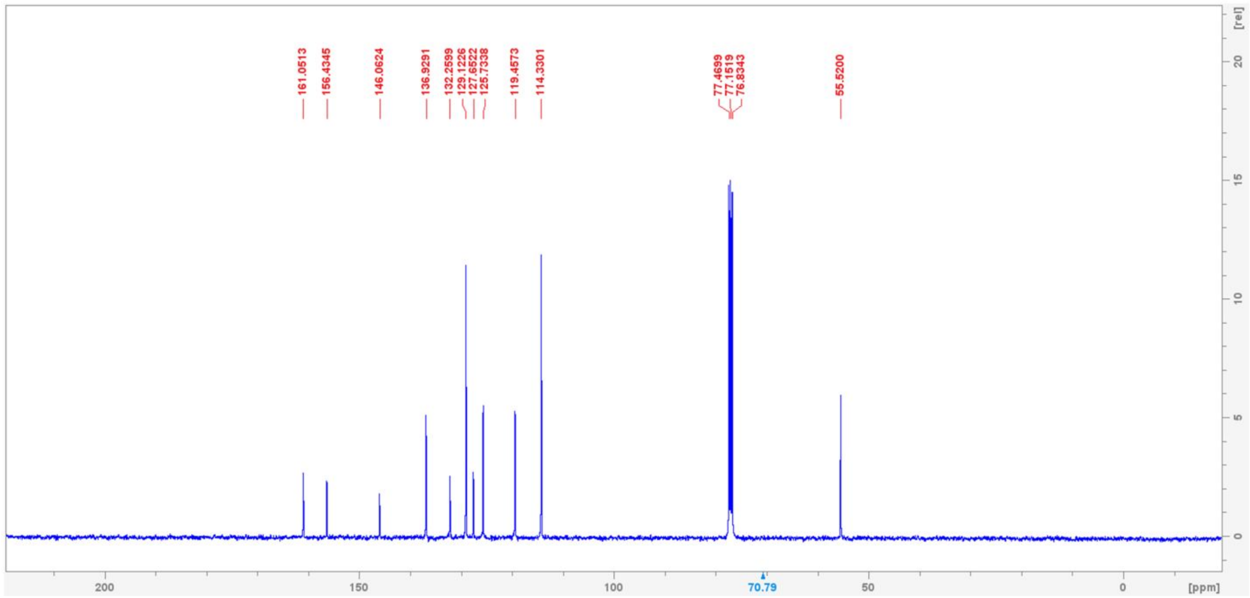

Figure S2. ^{13}C NMR ($CDCl_3$, 100.7 MHz) spectrum of 2,9-di-(*p*-methoxyphenyl)-1,10-phenanthroline.

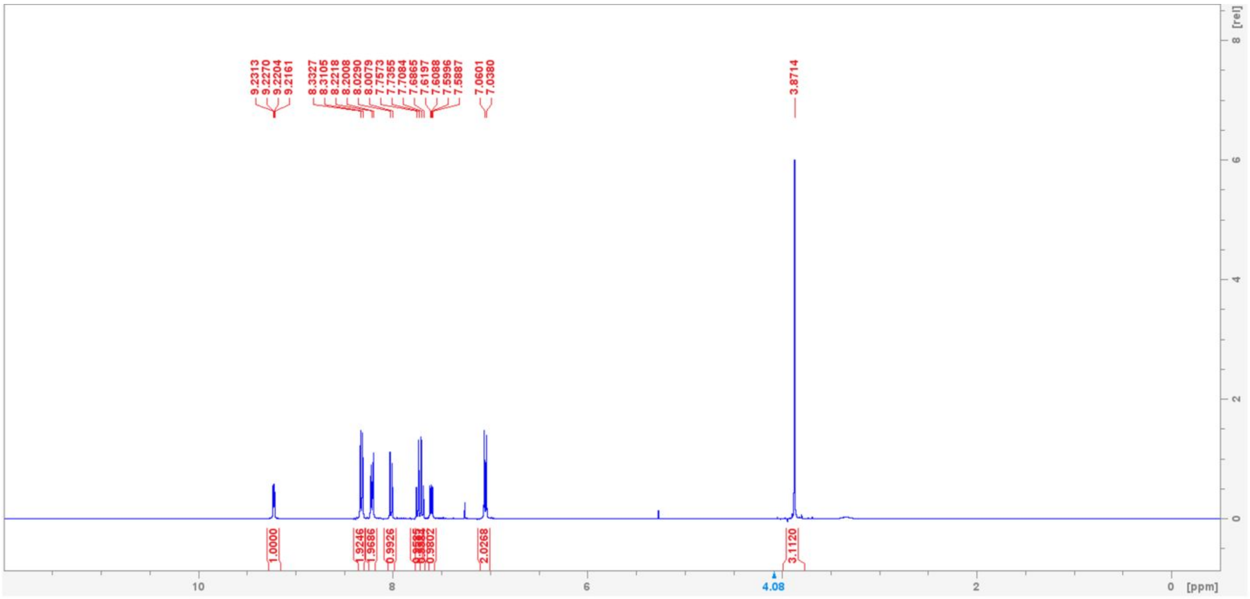

Figure S3. ^{1}H NMR ($CDCl_3$, 400 MHz) spectrum of 2-(*p*-methoxyphenyl)-1,10-phenanthroline.

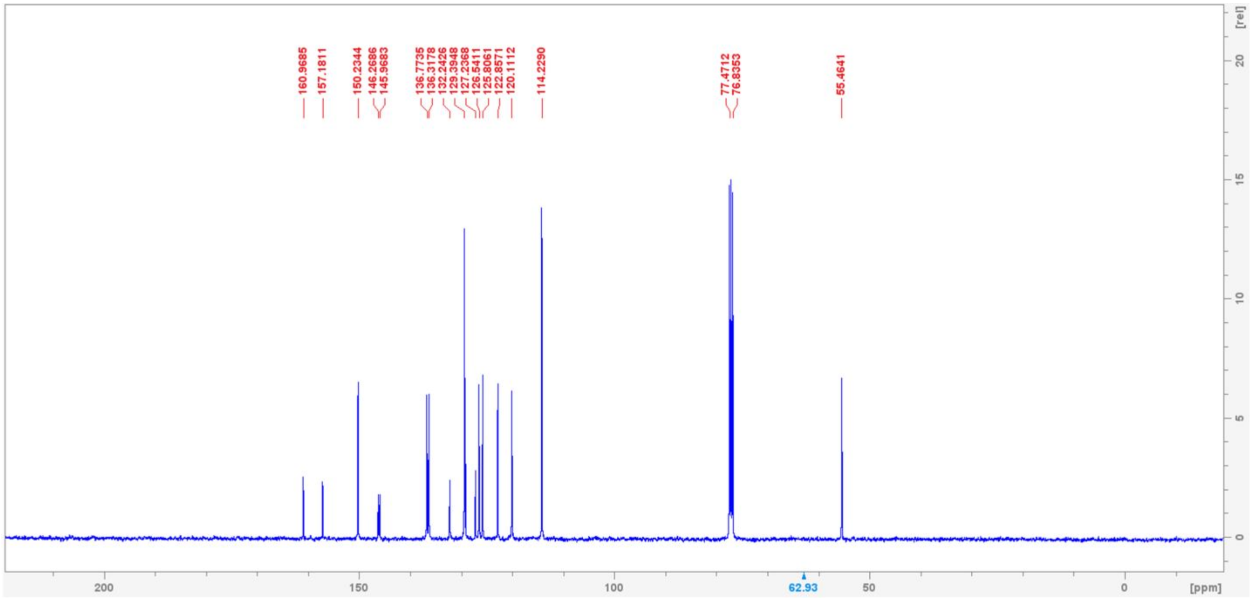

Figure S4. ^{13}C NMR ($CDCl_3$, 100.7 MHz) spectrum of 2-(*p*-methoxyphenyl)-1,10-phenanthroline.

UV-Vis Data.

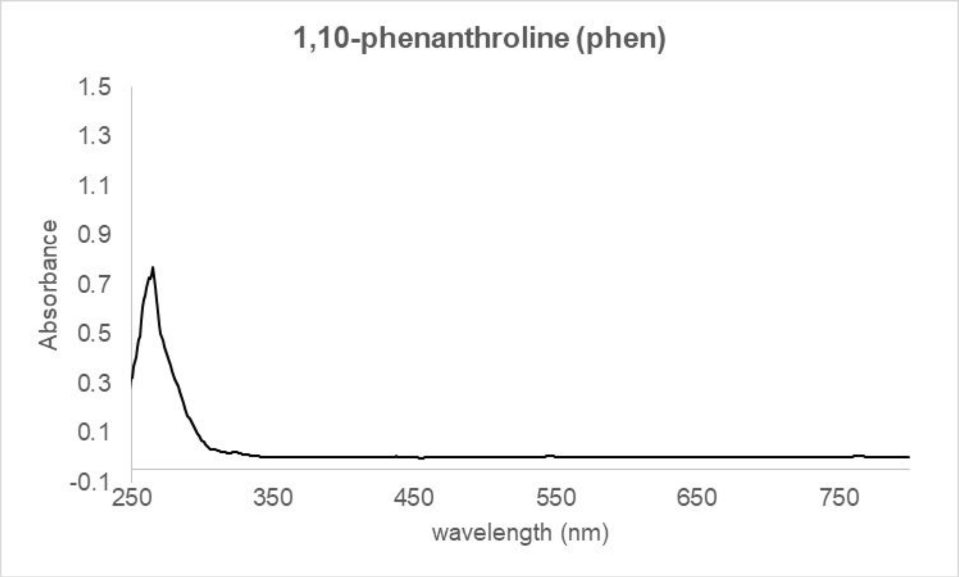

Figure S5. UV-Vis spectrum of 1,10-phenanthroline (1.6×10^{-5} M) in CH_2Cl_2.

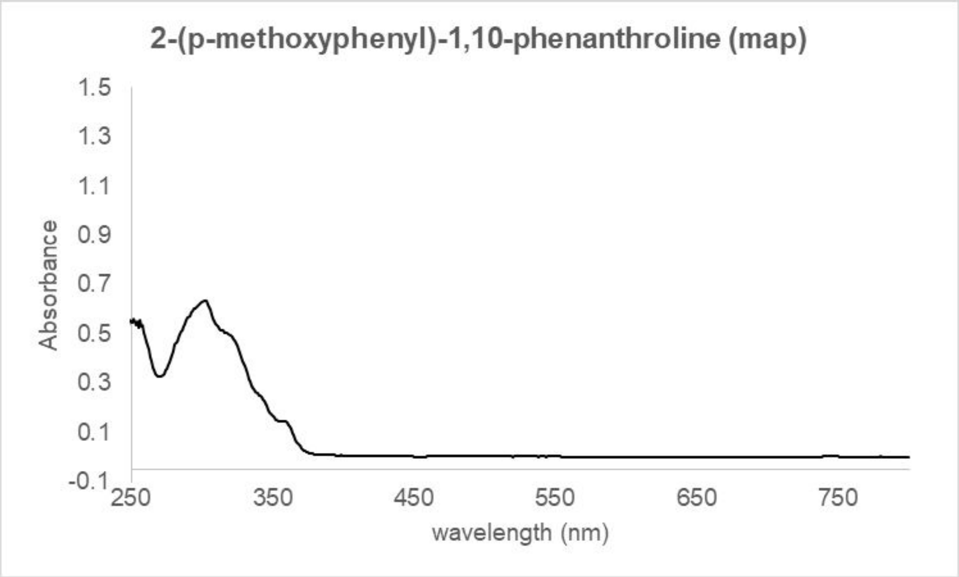

Figure S6. UV-Vis spectrum of 2-(*p*-methoxyphenyl)-1,10-phenanthroline (1.6×10^{-5} M) in CH_2Cl_2.

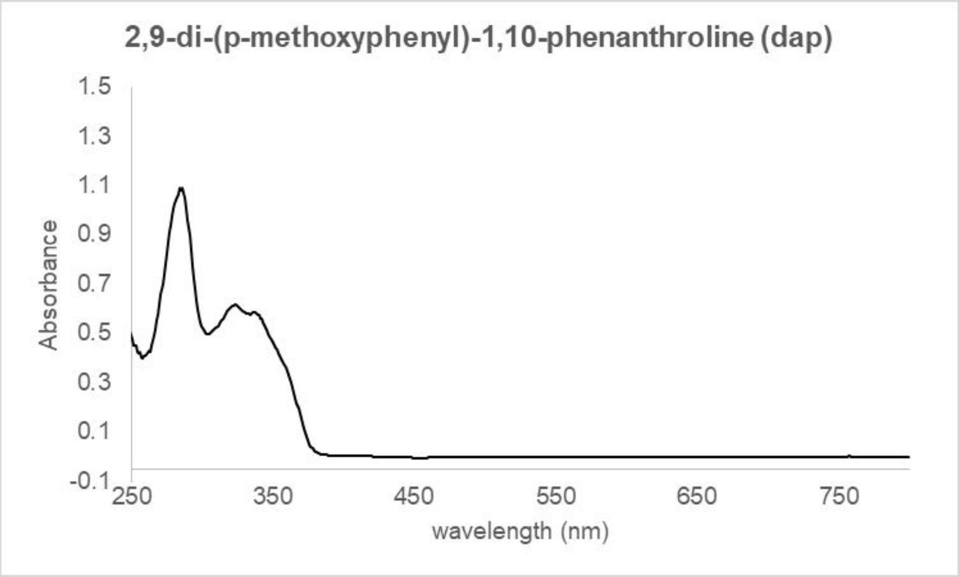

Figure S7. UV-Vis spectrum of 2,9-di-(*p*-methoxyphenyl)-1,10-phenanthroline (1.6×10^{-5} M) in CH_2Cl_2.

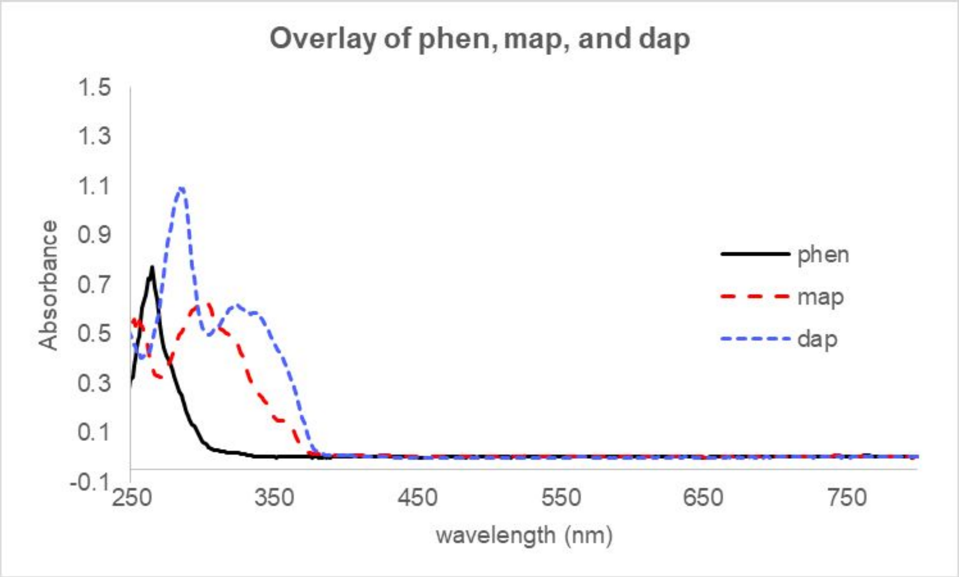

Figure S8. Overlaid UV-Vis spectra of 1,10-phenanthroline (phen), 2-(*p*-methoxyphenyl)-1,10-phenanthroline (map), and 2,9-di-(*p*-methoxyphenyl)-1,10-phenanthroline (dap) (concentrations are 1.6×10^{-5} M) in CH_2Cl_2.

GC-MS Data.

Data were collected using an Agilent 8890/5977C GC/MS configured for automatic injection of liquid samples. The samples were injected as CH_2Cl_2 solutions.

Column characteristics: Agilent HP-5 MS UI (30 m x 0.250 mm x 0.25 µm column dimensions).

Method 1 specifications: Inlet temperature: 250 °C. Oven temperatures: Initial, 2 min hold at 100°C. Ramp (8 min): 25°C/min. Final, 15 min hold at 300°C

Method 2 specifications: Inlet temperature: 250 °C. Oven temperatures: Initial, 2 min hold at 100°C. Ramp (8 min): 25°C/min. Final, 45 min hold at 300°C

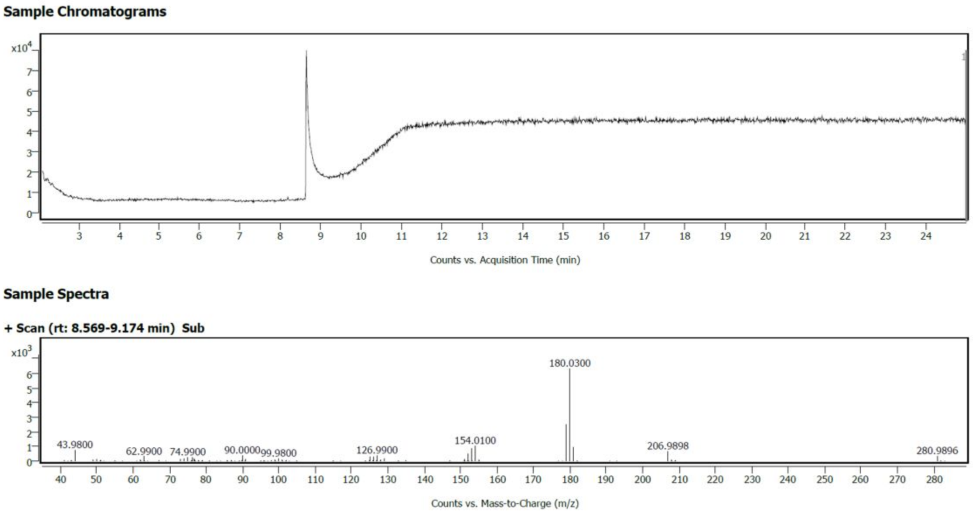

Figure S9. Gas chromatogram (top trace) and associated MS spectrum of 1,10-phenanthroline using Method 1.

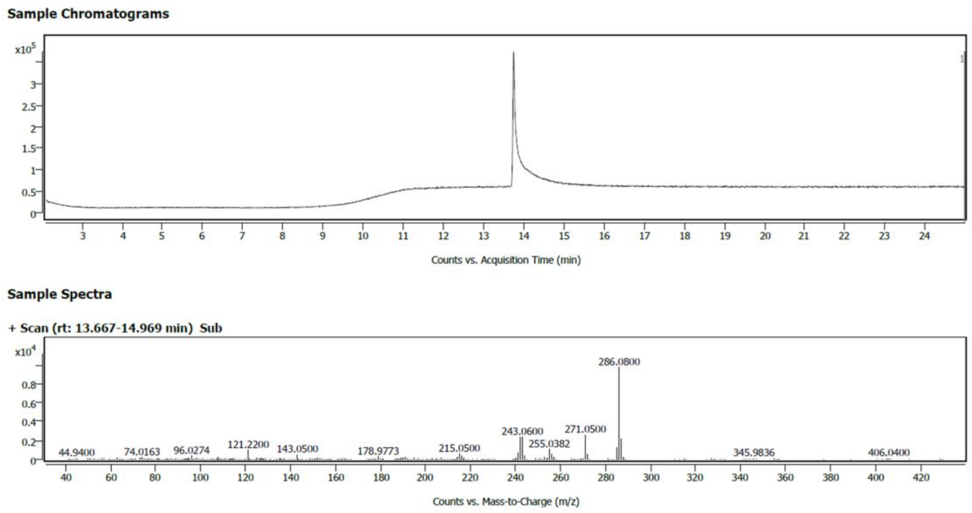

Figure S10. Gas chromatogram (top trace) and associated MS spectrum of 2-(*p*-methoxyphenyl)-1,10-phenanthroline using Method 1.

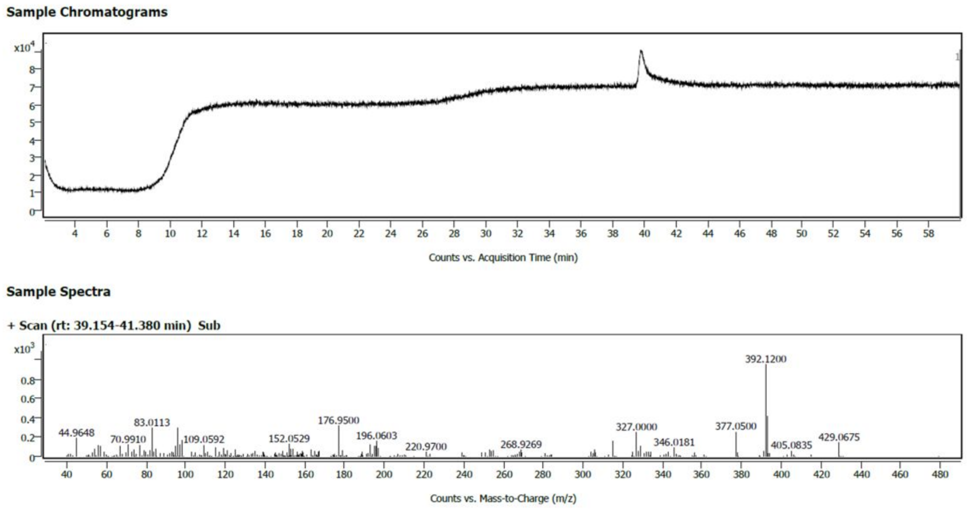

Figure S11. Gas chromatogram (top trace) and associated MS spectrum of 2,9-di-(*p*-methoxyphenyl)-1,10-phenanthroline using Method 2.

Attempted Arylation with Dimesitylmagnesium. In the glovebox, 0.265 g (0.0109 mol) of Mg turnings were added to a 50 mL round-bottom flask equipped with a stirbar. Approximately 15 mL of Et_2O was added, followed by 1.5 mL (0.0098 mol) of 2-bromomesitylene all at once. The mixture was stirred overnight, during which time the Mg was almost entirely consumed. To the resulting mixture, 1.8 mL (0.021 mol) of 1,4-dioxane was added, causing the rapid precipitation of gray solids. This was filtered through a Celite pad in a frit, and the contents of the flask were rinsed with several washes of Et_2O. The filtrate was transferred to a vial for storage to await titration. In the glovebox, 0.0349 g of (-)-menthol and 2 mg phenanthroline (as a titration indicator) were added to a 20 mL vial equipped with a stirbar. Approximately 5 mL of THF was used to dissolve the mixture. To the resulting stirring solution, the dimesitylmagnesium solution was added dropwise until a pink color was achieved at the endpoint (0.75 mL, corresponding to an organometallic mesityl (i.e. active Brønsted base) concentration of 0.30 M (or a molarity of dimesitylmagnesium of 0.15 M). Finally, to a separate 20 mL vial equipped with a stirbar in the glovebox, 0.177 g (0.000982 mol) of 1,10-phenanthroline was added. This was mixed with 5 mL of the dimesitylmagnesium solution (0.0015 mol mesityl), forming a dark brown suspension. An aliquot was taken from the stirring mixture after 3 days, indicating no reaction (workup described next). Finally, after 8 days total, the remaining reaction mixture was quenched with 4 mL NH_4Cl (1 M aqueous solution). The mixture was extracted with 3 x 5 mL CH_2Cl_2. A small portion of the organic solution was stirred over 86 mg of MnO_2 and stirred overnight. This aliquot mixture was filtered through Celite and evaporated to dryness to yield 28 mg, which was analyzed by ^1H NMR and showed primarily 1,10-phenanthroline (only traces of possible reactivity were evident).

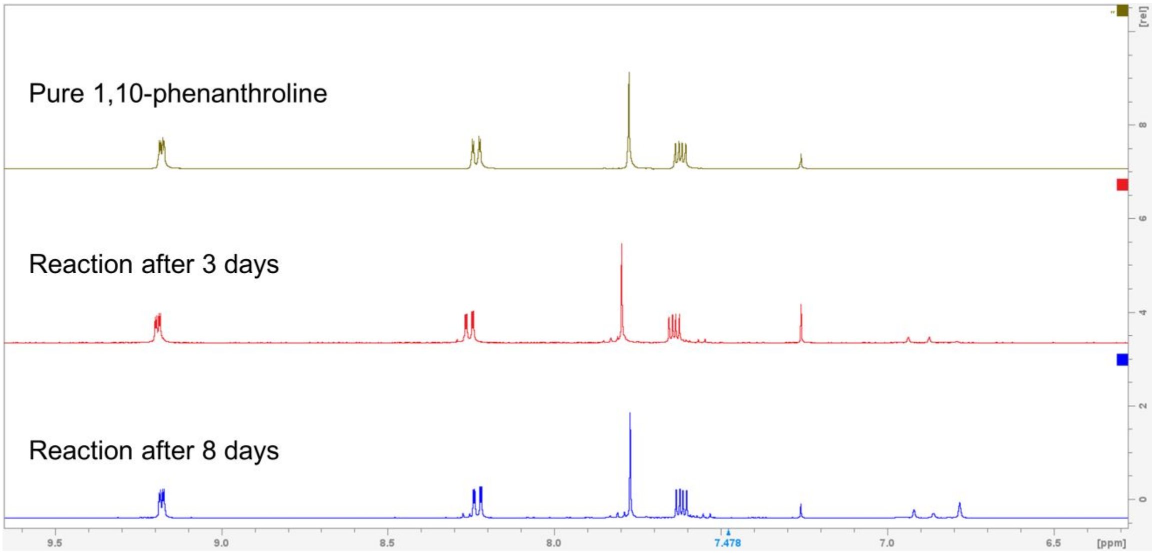

Figure S12. Comparison of the aromatic region of the ^1H NMR spectra for pure 1,10-phenanthroline (top) the quenched aliquot of the mixture with dimesitylmagnesium after 3 days (middle) and the quenched bulk after 8 days (bottom).

Successful Arylation with Di(*o*-tolyl)magnesium. In the glovebox, 0.336 g (0.0138 mol) of Mg turnings were added to a 50 mL round-bottom flask equipped with a stirbar. Approximately 15 mL of Et_2O was added, followed by gradual, portionwise addition of 1.5 mL (0.0125 mol) of 2-bromotoluene. Eventually, the Mg was almost entirely consumed. To the resulting mixture, 0.6 mL (0.0070 mol) of 1,4-dioxane was added, causing the rapid precipitation of gray solids. This was filtered through a Celite pad in a frit, and the contents of the flask were rinsed with several washes of Et_2O. The filtrate was transferred to a vial for storage to await titration. In the glovebox, 0.0685 g of (-)-menthol and 4 mg phenanthroline (as a titration indicator) were added to a 20 mL vial equipped with a stirbar. Approximately 5 mL of THF was used to dissolve the mixture. To the resulting stirring solution, the di(*o*-tolyl)magnesium solution was added dropwise until a pink color was achieved at the endpoint (0.98 mL, corresponding to an organometallic *o*-tolyl (i.e. active Brønsted base) concentration of 0.45 M (or a molarity of di(*o*-tolyl)magnesium of 0.22 M). Finally, to a separate 20 mL vial equipped with a stirbar in the glovebox, 0.051 g (0.00028 mol) of 1,10-phenanthroline was added. This was mixed with 0.92 mL of the di(*o*-tolyl)magnesium solution (0.00041 mol *o*-tolyl), forming a dark red suspension. An aliquot was taken from the stirring mixture after 1 day, indicating a complete reaction (workup described next). Finally, after 25 days, the remaining reaction mixture was quenched with 4 mL NH_4Cl (1 M aqueous solution). The mixture was extracted with 3 x 3 mL CH_2Cl_2. The organic solution was combined with 87 mg of MnO_2 and stirred overnight. This mixture was filtered through Celite and evaporated to dryness to yield 71 mg. This was purified by column chromatography (eluent: 2:1 EtOAc:hexanes). R_f (2:1 EtOAc:hexane) = 0.5. Yield: 0.026 g (34 % yield) with NMR data consistent with literature.[1]

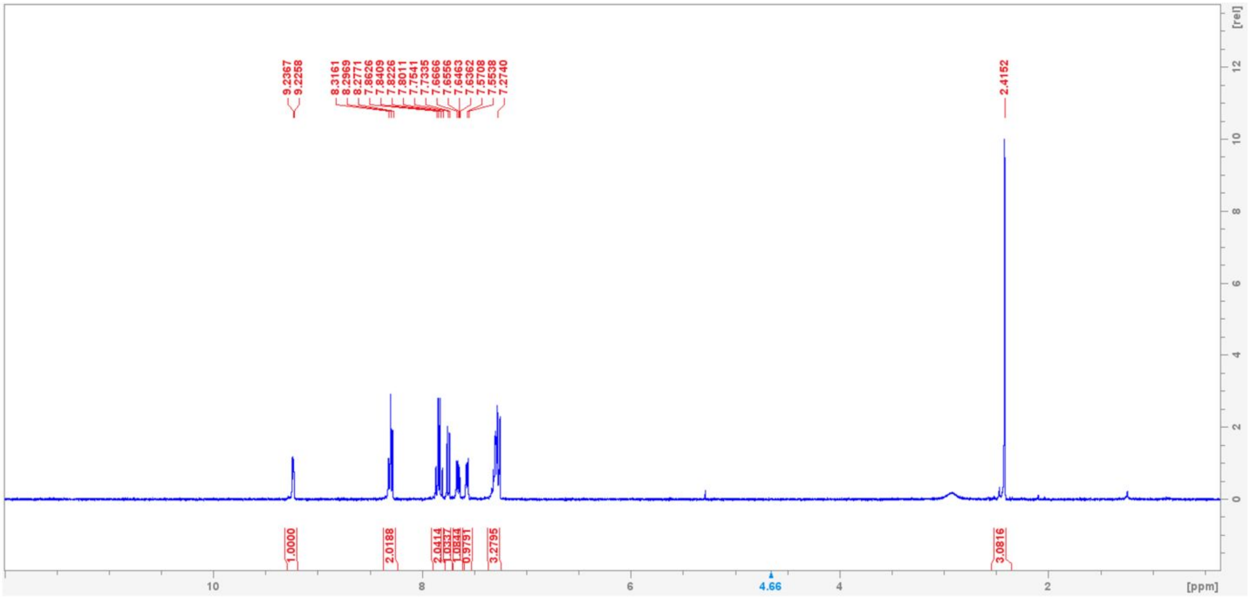

Figure S13. ^1H NMR ($CDCl_3$, 400 MHz) spectrum of 2-(*o*-tolyl)-1,10-phenanthroline.

Arylation with (*p*-methoxyphenyl)MgBr in THF. To a 250 mL Schlenk tube, Mg turnings (0.300 g, 0.0123 mol) were added, along with a stir bar. The flask was heat-gunned under vacuum, then backfilled with N_2 and allowed to cool to room temperature. Using a syringe, 24 mL of THF was added. Next, 1.4 mL of 4-bromoanisole was added all at once using a syringe. The mixture was stirred vigorously until the mixture was brown in color and most of the Mg turnings were obviously consumed. A separate 250 mL Schlenk tube was charged with 1,10-phenanthroline (1.36 g, 0.00755 mol) and a stirbar. This flask was evacuated for 2 hr on the Schlenk line. Then, it was backfilled with N_2 and 10 mL of THF was added via syringe. The THF solution of (*p*-methoxyphenyl)MgBr was added via cannula transfer dropwise over 5-10 min to the phenanthroline solution. The resulting dark red suspension was stirred at RT for 4 days. After this time, NH_4Cl (1.5 g, 0.028 mol) in 20 mL H_2O was used to quench the reaction. The yellow mixture was concentrated via rotary evaporation, then 50 mL CH_2Cl_2 and 25 mL H_2O was added. The aqueous layer was extracted with 2 x 25 mL CH_2Cl_2. The combined organic layers were stirred over MnO_2 (6.27 g, 0.0721 mol). After 4 days, this was filtered through Celite, and an aliquot was taken for a ^1H NMR of the crude mixture, which showed 4-(*p*-methoxyphenyl)-1,10-phenanthroline as a major product (see Figure S14, bottom).

Arylation with (*p*-methoxyphenyl)$_2$Mg in THF. A 25 mL Schlenk tube was charged with 1,10-phenanthroline (0.107 g, 0.000594 mol) and a stirbar. This flask was evacuated for 1 hr on the Schlenk line. Then, it was backfilled with N_2 and 10 mL of THF was added via syringe. To this, 2.9 mL of dianisylmagnesium (0.51 N in Et_2O, prepared and titrated against (-)-menthol as described in the main text, 0.0015 mol anisyl) was added all at once. The solvent composition is 3.4 : 1 THF:Et_2O by volume. The resulting dark red suspension was stirred at RT for 2 days. After this time, NH_4Cl (0.169 g, 0.00316 mol) in 1 mL H_2O was used to quench the reaction. The yellow mixture was concentrated via rotary evaporation which was extracted with 3 x 2 mL CH_2Cl_2. The combined organic layers were stirred over MnO_2 (0.50 g, 0.0058 mol). After 1 day, this was filtered through Celite, and an aliquot was taken for a ^1H NMR of the crude mixture. This showed a 1.6 : 1 ratio of 4-(*p*-methoxyphenyl)-1,10-phenanthroline (4-map) to 2-(*p*-methoxyphenyl)-1,10-phenanthroline (2-map), see Figure S14, middle. This was purified by column chromatography (initial eluent: 2:1 EtOAc:hexanes, final eluent: 95:5 CH_2Cl_2:MeOH). Yield: 86 mg 4-(*p*-methoxyphenyl)-1,10-phenanthroline (contaminated with 8% of unreacted 1,10-phenanthroline) with NMR data that is consistent with literature[2] (see Figure S14, top).

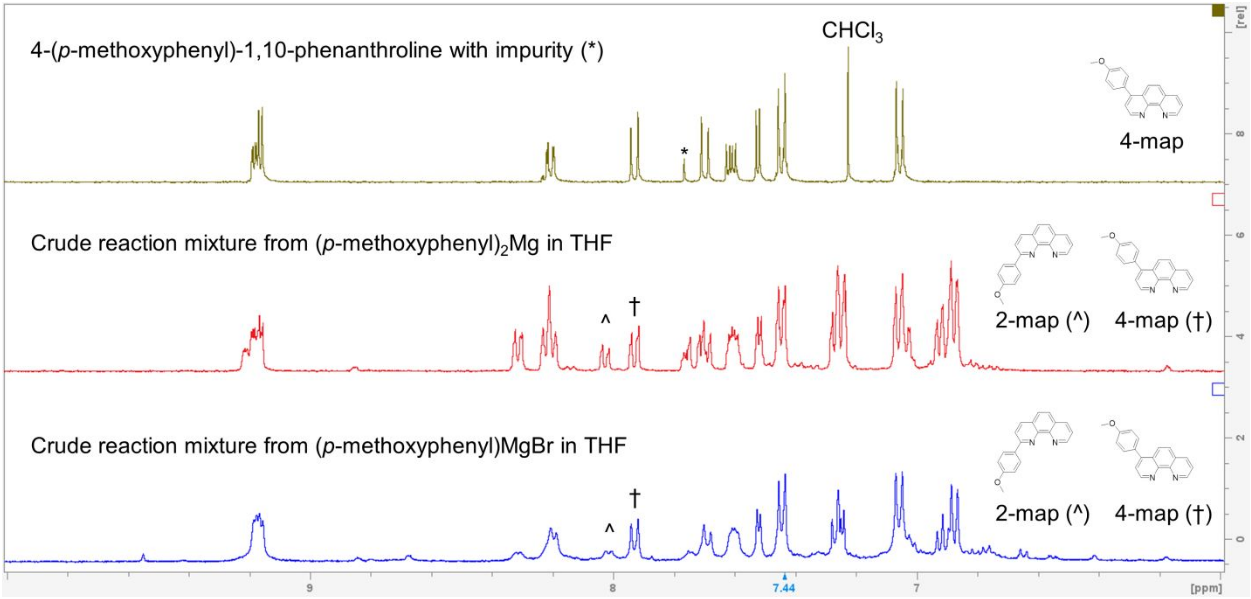

Figure S14. Comparison of the aromatic region of the ^{1}H NMR spectra of 4-(*p*-methoxyphenyl)-1,10-phenanthroline (top) the crude mixture from (*p*-methoxyphenyl)$_2$Mg in THF (middle) and the crude mixture from (*p*-methoxyphenyl)MgBr in THF (bottom).

References.

1. Colombo, A.; Ossola, R.; Magni, M.; Roberto, D.; Jacquemin, D.; Castellano, C.; Demartin, F.; Dragonetti, C. "Intriguing C–H···Cu Interactions in Bis-(Phenanthroline)Cu(I) Redox Mediators for Dye-Sensitized Solar Cells." *Dalton Trans.* **2018**, 47 (4), 1018–1022. https://doi.org/10.1039/C7DT04045H.

2. Laemmel, A.-C.; Collin, J.-P.; Sauvage, J.-P.; Accorsi, G.; Armaroli, N. "Macrocyclic Complexes of [Ru(N-N)2]2+ Units [N-N = 1,10 Phenanthroline or 4-(p-Anisyl)-1,10-Phenanthroline]: Synthesis and Photochemical Expulsion Studies." *Eur. J. Inorg. Chem.,* **2003,** 2003 (3), 467–474. https://doi.org/10.1002/ejic.200390066.
